# Cariprazine in First-Episode Psychosis with Comorbid Cannabis Use: A Case Series of Eight Patients from Two Greek Early Intervention Units

**DOI:** 10.3390/brainsci16080787

**Published:** 2026-07-26

**Authors:** Evangelos Ntouros, Despina-Christina Partsanaki, Stefanos Dimitrakopoulos, Elena Ioanna Nazlidou, Vasilis-Ilias Spatharas, Venetsanos Mavreas, Vasilis P. Bozikas

**Affiliations:** 1 Association for Regional Development and Mental Health (EPAPSY), 15124 Athens, Greece; entourob@auth.gr (E.N.); d.partsanaki@epapsy.gr (D.-C.P.); s.dimitrakopoulos@epapsy.gr (S.D.); e.nazlidou@epapsy.gr (E.I.N.); v.spatharas@epapsy.gr (V.-I.S.); v.mavreas@epapsy.gr (V.M.); 2 2nd Department of Psychiatry, School of Medicine, Aristotle University of Thessaloniki, 56430 Thessaloniki, Greece

**Keywords:** first-episode psychosis, cannabis, cariprazine, dual diagnosis, early intervention, PANSS, case series

## Abstract

**Highlights:**

**What are the main findings?**

- Over six months, cariprazine was associated with lower PANSS positive-symptom and disorganization scores in eight first-episode psychosis patients with comorbid cannabis use.
- Cannabis-use frequency decreased in seven of eight patients, with two achieving complete cessation; no causal inference can be drawn given the uncontrolled naturalistic design.

**What are the implications of the main findings?**

- The D3-preferential pharmacological profile of cariprazine may offer clinical advantages in the dual disorder of psychosis and cannabis use, warranting controlled investigation.
- Early intervention units represent an optimal setting for recruiting this underrepresented population in future prospective studies.

**Abstract:**

**Background/Objectives:** Cannabis use is prevalent in first-episode psychosis (FEP) and is associated with poor clinical outcomes. Cariprazine, a dopamine D3/D2 partial agonist with preferential D3 affinity, may offer clinical advantages for both psychotic symptoms and comorbid substance use; however, naturalistic data in FEP patients with active cannabis use are lacking. **Methods:** We conducted a retrospective naturalistic case series of eight FEP patients with active cannabis use treated with cariprazine for six months across two Greek Early Intervention Units (PNOES Athens and Thessaloniki, EPAPSY). Psychotic symptoms were assessed using the Positive and Negative Syndrome Scale (PANSS) Five-Factor (Marder) model and cannabis use using the Cannabis Experience Questionnaire (CEQ) at baseline and six months. Analyses were descriptive, with exploratory Wilcoxon signed-rank tests. **Results:** At six months, lower mean PANSS scores were observed for positive symptoms (12.88 vs. 9.50), disorganization (11.38 vs. 9.13) and, to a lesser degree, anxiety/depression (9.38 vs. 8.50). Negative symptoms changed little (16.13 vs. 15.00), and hostility was essentially unchanged (10.63 vs. 11.63). Only the reduction in positive symptoms reached nominal significance (*p* = 0.047; *d*_z_ = −1.05), which did not survive correction for multiple comparisons. Cannabis-use frequency decreased in seven of eight participants, with two reporting complete cessation. **Conclusions:** These preliminary, hypothesis-generating observations suggest that cariprazine may warrant further study in FEP with comorbid cannabis use. The findings cannot be interpreted as evidence of efficacy given the small, uncontrolled, diagnostically heterogeneous sample.

## 1. Introduction

Early intervention in the psychosis paradigm has been expanding in recent years, demonstrating improved symptom management, reduced hospitalization rates, and better social integration in early-onset psychosis, particularly First-Episode Psychosis (FEP) [1]. It involves antipsychotic medication combined with psychosocial interventions delivered by a multidisciplinary team; patients receiving early intervention report better quality of life, higher functioning, and improved treatment compliance [2].

The comorbidity of substance use disorder (SUD) with psychosis, referred to as dual disorder (DD), significantly increases morbidity and mortality compared with psychosis alone, with cannabis being the most frequently used substance [3]. In genetically vulnerable individuals, cannabis may precipitate FEP by disrupting dopaminergic and endocannabinoid signaling. Its use is linked to an earlier age of psychosis onset and poorer clinical outcomes, with an estimated prevalence of cannabis use of 33.7% in FEP patients [3,4]. Regular use confers a 3–5-fold increase in the risk of developing a psychotic disorder in a dose-dependent fashion [3]. Continued use following FEP worsens prognosis and increases relapse risk [4,5].

Cariprazine is a third-generation atypical antipsychotic acting as a partial agonist at D3 and D2 dopamine receptors with preferential D3 affinity, as well as affinity for serotonin 5-HT1A and 5-HT2A receptors [6]. A systematic review and meta-analysis has demonstrated its transdiagnostic efficacy across positive, negative, manic, depressive, and cognitive symptom domains [7]. A six-month observational study in patients with established schizophrenia and cannabis use disorder reported improvements in both psychotic and addiction-related measures, tentatively attributed to its D3-preferential binding profile [8].

Beyond its antipsychotic activity, cariprazine’s preferential affinity for the dopamine D3 receptor provides a plausible rationale for effects on comorbid cannabis use. D3 receptors are concentrated in mesolimbic reward circuitry, including the ventral striatum and nucleus accumbens, where they modulate motivation, incentive salience, and drug-related craving [6]. D3 receptor availability is upregulated by chronic exposure to substances of abuse, and D3 partial agonism or antagonism can attenuate drug-seeking and cue-induced craving. Because most antipsychotics occupy D3 receptors minimally at clinical doses, cariprazine’s D3-preferential partial agonism is mechanistically distinctive and has been proposed as a means of simultaneously addressing psychotic symptoms and the reward-related processes that sustain substance use in dual disorder [6,8]. This provides the pharmacological premise examined, at a descriptive level, in the present series.

This case series reports preliminary naturalistic observations on cariprazine treatment in FEP with comorbid active cannabis use. In contrast to existing reports, which concern established or chronic schizophrenia [8] or the general clinical impact of cannabis in psychosis [5,9], and to trial-level efficacy summaries [7], we provide structured, individual-level data from an early-intervention setting for a population in which such data are currently lacking.

Accordingly, the objectives of this case series were threefold: (i) to describe changes in psychotic symptomatology, assessed using the PANSS five-factor (Marder) model, over six months of cariprazine treatment in patients with FEP and comorbid active cannabis use; (ii) to describe concurrent changes in cannabis use and use frequency, assessed using the CEQ, over the same period; and (iii) to document the individual clinical trajectories of these patients within routine early-intervention care, in order to generate hypotheses for future controlled research in this underrepresented population. Given the exploratory, uncontrolled design and the small sample, no efficacy hypotheses were formally tested.

## 2. Materials and Methods

This was a retrospective naturalistic case series of consecutive patients diagnosed with FEP and concomitant cannabis use who were treated with cariprazine for six months between 2023 and 2025. Here, “naturalistic” denotes that all treatment decisions—including the choice of cariprazine and its dosing—were made by the treating clinicians as part of routine care, without protocol-driven randomization, fixed dosing, or standardized co-interventions. The sample comprised eight patients receiving care at the Early Intervention Units for Psychosis in Athens and Thessaloniki (PNOES Ath and PNOES SKG) of EPAPSY (Association for Regional Development and Mental Health). The inclusion criteria were: (a) clinical diagnosis of FEP, (b) documented current cannabis use at presentation, and (c) clinical initiation of cariprazine as part of routine care. The decision to prescribe cariprazine was based solely on the treating psychiatrist’s clinical assessment and was not part of any predefined research protocol. No structured psychosocial intervention was standardized across participants, although all patients received standard psychosocial support within the Early Intervention Unit’s multidisciplinary care. Table 1 presents the sociodemographic, clinical, and outcome data for each participant.

The sample included four men and four women, with a mean age of 26.75 years. The diagnoses (ICD-10) included schizophrenia (F20, *n* = 4), schizoaffective disorder (F25, *n* = 1), bipolar disorder (F31, *n* = 1), brief psychotic disorder (F23, *n* = 1), and unspecified functional affective psychosis (F39, n = 1). The mean Duration of Untreated Psychosis (DUP) was 18.5 weeks. All participants received cariprazine at doses ranging from 1.5 to 4.5 mg/day; one participant (patient 2) received adjunctive olanzapine 20 mg concurrently from the third month onward.

Antipsychotic symptom outcomes were measured using the Positive and Negative Syndrome Scale (PANSS) at baseline and six months, reported using the Five-Factor (Marder) model encompassing positive symptoms, negative symptoms, disorganization, anxiety/depression, and hostility. Cannabis use and frequency were assessed using items 15.4 and 15.9 of the Greek-validated Cannabis Experience Questionnaire (CEQ) at baseline and six months [10].

Given the small sample size, analyses were primarily descriptive, with continuous outcomes summarized as means and changes from baseline. As exploratory analyses, paired baseline-to-six-month comparisons for each PANSS factor were examined using the Wilcoxon signed-rank test, with 95% confidence intervals for the mean change and paired effect sizes (Cohen’s *d*_z_); because formal tests of normality are uninformative at this sample size, a non-parametric test was pre-selected. A sensitivity analysis repeated the positive-symptom comparison after excluding the participant who received adjunctive olanzapine. Analyses were performed in Python 3 (SciPy). Given the small sample and multiplicity, inferential results are interpreted as exploratory and hypothesis-generating, and no causal inference is drawn.

This retrospective observational case series was conducted in accordance with the Declaration of Helsinki and approved by the Human Investigation Committee (IRB) of EPAPSY; written informed consent was obtained from all participants. During preparation of this manuscript, the authors used Claude (Anthropic) for language editing and to assist with data-table reconciliation and statistical computation; all outputs were reviewed and verified by the authors, who take full responsibility for the content.

## 3. Results

Table 2 presents individual raw scores for the Five-Factor PANSS and CEQ items at baseline and six months. At the group level, lower mean scores were observed at six months for positive symptoms (12.88 vs. 9.50) and disorganization (11.38 vs. 9.13), with a smaller reduction in anxiety/depression (9.38 vs. 8.50). Negative symptoms changed little (16.13 vs. 15.00), and hostility was essentially unchanged (10.63 vs. 11.63).

In exploratory Wilcoxon signed-rank tests, only the reduction in positive symptoms reached nominal significance (mean change −3.38, 95% CI −6.05 to −0.70; *p* = 0.047; *d*_z_ = −1.05). Changes in disorganization (−2.25, 95% CI −5.17 to 0.67; *p* = 0.172), negative symptoms (−1.12, 95% CI −7.48 to 5.23; *p* = 0.547), anxiety/depression (−0.88, 95% CI −4.60 to 2.85; *p* = 0.641), and hostility (+1.00, 95% CI −3.07 to 5.07; *p* = 0.812) were not significant. The positive-symptom result did not survive correction for five comparisons and is reported as exploratory. In a sensitivity analysis excluding the participant receiving adjunctive olanzapine (patient 2), the positive-symptom reduction was of similar magnitude (mean change −3.57, 95% CI −6.72 to −0.42) but no longer reached nominal significance (*p* = 0.062).

All eight participants reported active cannabis use at baseline. At baseline, four participants (50%) used cannabis more than once a week, two (25%) a few times a year, one (12.5%) a few times a month, and one (12.5%) daily. At six months, cannabis-use frequency had decreased in seven of eight participants and was unchanged in one, with no participant increasing use; two participants (25%) achieved complete cessation. The six-month distribution was four participants (50%) using a few times a month, one (12.5%) a few times a year, one (12.5%) more than once a week, and two (25%) reporting no use.

## 4. Discussion

The present report describes preliminary naturalistic observations from an exploratory case series of eight FEP patients with comorbid cannabis use treated with cariprazine for six months within early intervention units.

The key contribution of this case series is not confirmation of efficacy but the provision of individual-level clinical data from a specific and underrepresented population: FEP patients with active cannabis use receiving cariprazine within an early intervention framework. To our knowledge, structured naturalistic data from this population have not previously been reported. Although a prior six-month observational study examined 58 patients with established schizophrenia and cannabis use disorder, reporting improvements in both psychotic and addiction-related measures [8], the present observations extend the question to the first-episode phase, where illness chronicity and prior antipsychotic exposure are limited, and the therapeutic window for reducing cannabis use may be the widest. Small case reports and series likewise support cariprazine as a maintenance option following FEP, including when an initial antipsychotic has been poorly tolerated [11,12,13]; none, however, has focused specifically on comorbid active cannabis use at the individual level.

Lower PANSS scores at six months were observed in the positive-symptom and disorganization dimensions, with a smaller reduction in anxiety/depression. The small change in negative symptoms (mean difference 1.12 points) and the essentially unchanged hostility scores may reflect the diagnostic heterogeneity of the sample, the relatively short follow-up, or the characteristics of the specific patients included. In exploratory testing, only the positive-symptom reduction reached nominal significance, and this did not survive correction for multiple comparisons; these observations align broadly with the established antipsychotic profile of cariprazine across symptom domains [7].

Longitudinal data from larger cohort studies provide an important reference frame. Clausen et al. found that FEP patients who discontinued cannabis use showed substantially lower psychotic symptom levels at five-year follow-up [5]. Setíen-Suero et al. reported that cannabis cessation was associated with significantly better long-term outcomes in the PAFIP cohort (Programa Asistencial de las Fases Iniciales de Psicosis, Cantabria, Spain) [9]. An earlier report from the same group documented sex-related differences in clinical and neuropsychological outcomes associated with cannabis use in FEP [4]. Additionally, the CAFEPS study (Child and Adolescent First-Episode Psychosis Study) demonstrated that cannabis use at the time of the first episode adversely influenced psychopathology and short-term outcome in a pediatric-onset FEP sample [14]. These findings collectively underscore the clinical relevance of reducing cannabis use in FEP.

The observed reduction in cannabis-use frequency—seven of eight participants using less frequently at six months, including two who achieved complete cessation—is noted descriptively and cannot be attributed to cariprazine alone, as all participants received structured multidisciplinary psychosocial support within the early intervention units.

This study has several limitations. First, a naturalistic non-controlled design precludes any causal relationship between cariprazine and observed changes. Second, the small sample size and diagnostic heterogeneity substantially limit interpretability and generalizability. Third, cannabis use was assessed via self-report using the CEQ, introducing potential under-reporting bias. Fourth, the inferential analyses were exploratory and statistically underpowered; the single nominally significant result did not survive correction for multiple comparisons. Fifth, potential confounders—including concurrent psychosocial interventions, therapeutic alliance, natural illness course, concomitant medication, and concurrent life events—were not systematically controlled. Sixth, the six-month follow-up may be insufficient to detect long-term changes, particularly in negative symptoms. Future research should employ randomized or controlled designs with larger, diagnostically homogeneous samples, objective cannabis-use monitoring, and standardized psychosocial intervention protocols.

## 5. Conclusions

This exploratory naturalistic case series provides preliminary descriptive observations suggesting the possible clinical utility of cariprazine in FEP patients with comorbid cannabis use. Lower PANSS scores in positive symptoms and disorganization and a reduction in cannabis-use frequency were observed over six months, although these findings are not interpretable as evidence of efficacy given the study’s methodological constraints. The data are best understood as hypothesis-generating and as a contribution to the limited individual-level clinical literature on this population within an early intervention context. Controlled prospective studies are required to evaluate the therapeutic potential of cariprazine in this population.

## Figures and Tables

**Table 1:** Individual-level sociodemographic, clinical, and outcome data for each participant.

Pt.	Sex	Age	Employment	Dx (ICD-10)	DUP (wks)	Cariprazine (mg/day)	Adjunctive Antipsychotic	Cannabis Frequency Baseline	Cannabis Frequency 6 months
1	Female	32	Unemployed	F25	8	3	—	A few times/year	No use
2	Male	22	Private-sector employee	F20	12	3	Olanzapine 20 mg (from month 3)	>Once/week	A few times/month
3	Female	24	Student	F39	36	3	—	>Once/week	A few times/month
4	Male	19	Student	F20	58	1.5	—	>Once/week	A few times/month
5	Male	25	University student	F20	52	3	—	>Once/week	>Once/week
6	Male	32	Unemployed	F20	8	4.5	—	A few times/month	A few times/year
7	Female	29	Private-sector employee	F31	3	3	—	A few times/year	No use
8	Female	31	Private-sector employee	F23	8	3	—	>Once/week	A few times/month

**Note.** Pt. = patient number. Dx = ICD-10 diagnosis. DUP = duration of untreated psychosis. Cannabis-use frequency is the CEQ item-15.9 category. Sample: four men and four women; mean age 26.75 years; mean DUP 18.5 weeks. — = none.

**Table 2:** Individual and mean Five-Factor PANSS and CEQ item scores at baseline and six-month follow-up.

Pt.	Positive		Negative		Disorg.		Hostility		Anx/Dep		CEQ 15.4		CEQ 15.9	
	BL	6 m	BL	6 m	BL	6 m	BL	6 m	BL	6 m	BL	6 m	BL	6 m
**1**	9	12	16	16	9	10	10	14	7	11	2	1	4	6
**2**	8	6	22	14	12	10	6	6	7	5	2	2	1	3
**3**	13	6	16	17	15	11	17	16	7	9	2	2	2	3
**4**	18	14	24	16	17	9	11	13	12	6	2	2	2	3
**5**	22	16	15	29	11	13	13	24	15	13	2	2	2	2
**6**	13	7	16	11	13	7	9	5	9	6	2	2	3	4
**7**	8	6	13	6	6	6	9	5	11	5	2	1	4	6
**8**	12	9	7	11	8	7	10	10	7	13	2	2	2	3
**Mean**	**12.88**	**9.50**	**16.13**	**15.00**	**11.38**	**9.13**	**10.63**	**11.63**	**9.38**	**8.50**	**—**	**—**	**—**	**—**

Note. Pt. = patient number. Baseline (BL) and six-month (6 m) values are shown side by side for each measure. PANSS scores are Marder five-factor scores: Positive, Negative, Disorg. = disorganization, Hostility, Anx/Dep = anxiety/depression. CEQ = Cannabis Experience Questionnaire (Greek validation). Item 15.4 = cannabis use (1 = no; 2 = yes). Item 15.9 = frequency of use (1 = daily; 2 = more than once/week; 3 = a few times/month; 4 = a few times/year; 5 = only 1–2 times in total; 6 = no use); higher scores denote less frequent use. Two participants (patients 1 and 7) reported no use at six months (15.4 = 1; 15.9 = 6).

## Data Availability

Data supporting the reported results are not publicly available due to privacy and ethical restrictions regarding patient information.

## Abbreviations

**CEQ**	Cannabis Experience Questionnaire
**DD**	Dual Disorder
**DUP**	Duration of Untreated Psychosis
**FEP**	First-Episode Psychosis
**ICD-10**	International Classification of Diseases, 10th Revision
**IRB**	Institutional Review Board
**PANSS**	Positive and Negative Syndrome Scale
**PNOES**	Early Intervention Unit for Psychosis (Greek acronym)
**SUD**	Substance Use Disorder
